# Supplementary figures and images for: Diagnostic value of the molecular detection of Sarcoptes scabiei from a skin scraping in patients with suspected scabies
Source: PLoS Negl Trop Dis. 2020 Apr 7;14(4):e0008229. doi: 10.1371/journal.pntd.0008229 (PMC7164670; doi:10.1371/journal.pntd.0008229)

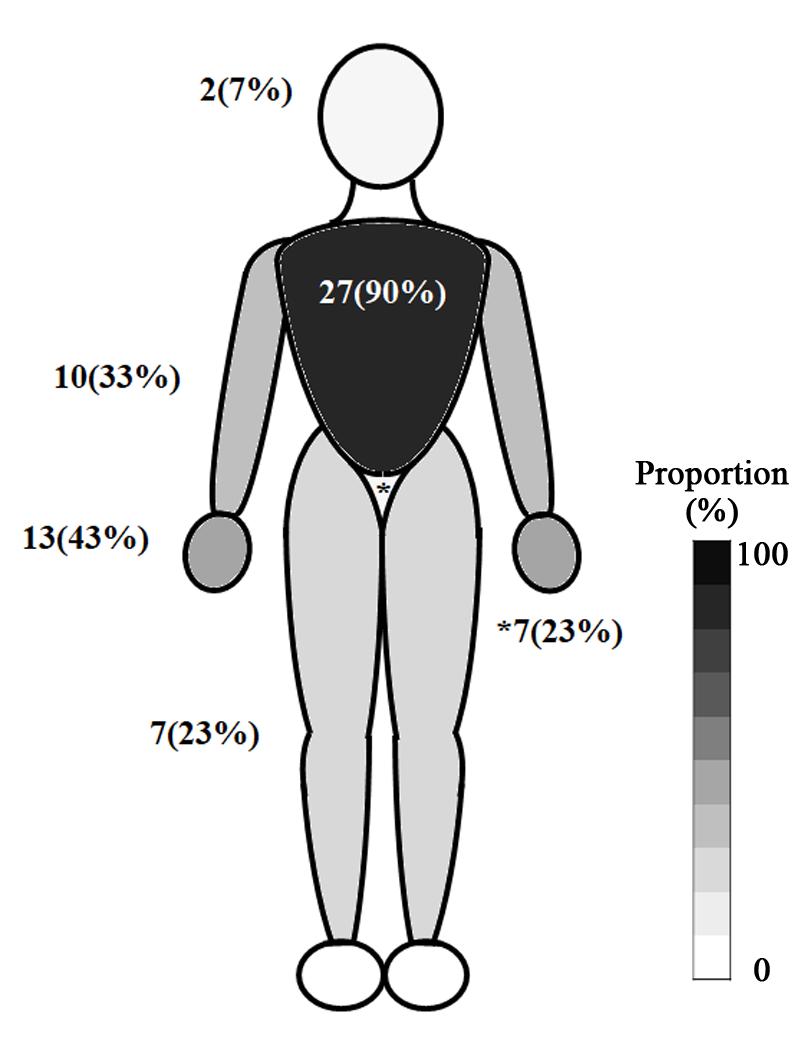

Supplement: S1 Fig — Percentage in the specific locations referred to the proportion of patients with scabies who presented skin lesion at that location. (TIF) [file pntd.0008229.s001.tif]
