# Supplementary material for: Diagnostic value of the molecular detection of Sarcoptes scabiei from a skin scraping in patients with suspected scabies
Source: PLoS Negl Trop Dis. 2020 Apr 7;14(4):e0008229. doi: 10.1371/journal.pntd.0008229 (PMC7164670; doi:10.1371/journal.pntd.0008229)
Supplement: Attachment — Submitted filename: Response_to_Reviewers.docx [file pntd.0008229.s002.docx]

Paul J. Brindley, PhD

Editor-in-Chief

PLOS Neglected Tropical Diseases

Date: 2 March 2020

**Re: PNTD-D-19-01821, entitled "Diagnostic value of the molecular detection of *Sarcoptes scabiei* from a skin scraping in patients with suspected scabies"**

Dear Brindley:

Thank you for the opportunity to revise our manuscript “Diagnostic value of the molecular

detection of *Sarcoptes scabiei* from skin scrapings in patients with suspected scabies”. We appreciate the reviewers’ helpful comments for correction or modification. We believe that the manuscript is substantially improved after making the suggested edits.

Following this letter are the reviewers’ comments with our responses, including how and where the text was modified. Changes made in the manuscript are highlighted in yellow.

We would be happy to make further corrections if necessary, and look forward to hearing from you soon.

Sincerely yours,

Sung-Han Kim, M.D.

Professor

Department of Infectious Diseases

Asan Medical Center

University of Ulsan College of Medicine

88, Olympic-ro 43-gil,

Songpa-gu, Seoul 05505, Republic of Korea

**Editor’s comment:**

**1. Line 36: Do not use abbreviations such as "exam"**

🡪 As the editor’s comments, we have corrected “exam” to “examination” as follows.

**“**We found that the diagnostic sensitivity of scabies PCR maintained between 86% and 80% as the level of diagnostic certainty by the IACS criteria decreased, while the diagnostic sensitivity of microscopic examinations decreased from 100% to 73% as the level of diagnostic certainty by the the IACS criteria decreased.**”**

**2. Line 38: Place a full stop at the end of the sentence**

🡪 As the editor’s comments, we have added a period at the end of the sentence as follows.

“Our data suggested that our in-house scabies PCR test was a useful adjunct in the improvement for the diagnosis of scabies by the consensus criteria.”

**3. Line 62: scabiei rather than scabies.**

🡪 As the editor’s comments, we have corrected “*Sarcoptes scabies*” to “*Sarcoptes scabiei*” as follows.

“PCR was performed on the skin scrapings to target the cytochrome c oxidase subunit 1 (cox1) gene of *Sarcoptes scabiei*.”

**4. Line 63-65: The sum of the patients mentioned is not 43 (see also lines 195-197)**

🡪 As the editor’s comments, “43” is a typo-error. “33” is correct. A total of 47 participants, 33 with suspected scabies, 10 with onychomycosis, and 4 healthy volunteers were included. Of these 33 patients with suspected scabies, 22 were classified as confirmed scabies, 2 as clinical scabies, 6 as suspected scabies, and 3 as no scabies. A total of 17 participants including 3 with no scabies, 10 with onychomycosis, and 4 healthy volunteers were classified as no scabies control. We corrected each number and revised the manuscript as follows.

Abstract-

“A total of 47 participants, 33 with suspected scabies, 10 with unrelated diseases, and 4 healthy volunteers were enrolled. Of the 33 patients, 22 were classified as confirmed scabies, 2 as clinical scabies, 6 as suspected scabies, and 3 as no scabies.”

Results-

“A total of 47 participants including 33 patients with suspected scabies and 14 participants with an unrelated disease (10 patients with onychomycosis and 4 healthy volunteers) were enrolled in this study. Of the 33 patients with suspected scabies, 22 were classified as confirmed scabies, 2 as clinical scabies, 6 as suspected scabies, and 3 as no scabies (2 as drug eruption, 1 as another superficial fungal infection).”

“Skin scraping specimens from 10 patients with onychomycosis, 4 healthy donors without any other dermatologic condition, and 3 classified as no scabies by the IACS criteria were all negative for the PCR test.”

**5. Line 83: Mention also the crusted scabies.**

🡪 As the editor’s comments, we have added some additional information at the Introduction section of the revised manuscript as follows.

“One of the most important risk factors for nosocomial outbreaks is the failure to recognize scabies in patients by attending clinicians. The crusted scabies has particularly atypical presentation making it difficult to diagnose and high risk of transmission, therefore it has been the index case in most recent outbreaks [4]. ”

**6. Line 90: Mention also the dermoscope**

🡪 As the editor’s comments, we have added some additional information at the Introduction section of the revised manuscript as follows.

“Microscopic examination of skin scrapings is widely used for the diagnosis of scabies, but it has a suboptimal sensitivity of only 50% [7]. Dermoscopy is also widely used, but it has a disadvantage in that it has low specificity and is affected by the dermatologist’s experience [7]. More sensitive and specific diagnostic tests are urgently required.

**7. Line 232: Mention the number as 13.**

🡪 As the editor’s comments, we have replaced “thirteen” with “13” as follows.

“^a^“No scabies” included 13 skin scrap specimens from patients with alternative diagnosis and four skin scrap specimens from healthy volunteers.”

**Reviewer #1’s comment:**

**1. Methods- Please add no and date of ethical approval.**
🡪 Per the reviewer’s comment, we have added number and date of ethical approval.

“The study’s approval was obtained from the Institutional Review Board of the Asan Medical Center (2018-0640).”

**2. Results- Are there any differences for the results according to gender or age? Are there any differences for the results according to the distribution of lesions (finger-webs, wrists, hands, axillae, gluteus, genitals, or breasts or may be mixed?)? Are there any differences for the results according to lesions (multiple small papules, nodules, vesicles, or excoriation or others ???)? Please add your results (Could be the reason for getting different results).**

🡪 We see the reviewer’s point. To determine whether there is a difference in the PCR sensitivity according to gender, age, location of skin lesions, and type of skin lesion, we performed the additional analysis. Of 30 patients with scabies, 17 were male and 13 were female. The scabies PCR sensitivity in males and females was 82% vs. 77% (*p*=0.53), respectively. Of 30 patients with scabies, 15 were ≥ 60 years old and the remaining 15 were under 60 years old, and their PCR sensitivity was 73% vs. 87%, respectively (*p*=0.33). Of 30 patients with scabies, 23 had papule and 7 had other skin lesions such as nodule and excoriated patches. The PCR sensitivity was 83% vs. 71%, respectively (*p*=0.60).

The torso (include groin) was the most common location in 27 patients (90%), followed by the extremities in 22 (73%) of the patients (S1 Fig). The scabies PCR sensitivities according skin lesions were 82% at extremities, 81% at torso, and 85% at extremities and torso, respectively (*p*=0.99)

In conclusion, there was no statistically significant difference in the PCR sensitivity according to gender, age, location of skin lesion, and type of skin lesion. We added this information in the Results section and the Discussion section of the revised manuscript as follows.

**S1 Fig. Distribution of skin lesions. Percentage in the specific locations referred to the proportion of patients with scabies who presented skin lesion at that location.**

Results-

“The torso was the most common location in 27 patients (90%), followed by the extremities in 22 (73%), ~~the groin in 9 (30%),~~ and the genitalia in 7 (23%) of the patients. Approximately 43% of patients with scabies had the skin lesion in the hands (S1 Fig).”

“Thus, the specificity of the scabies PCR in no scabies controls was of 100% (95% CI=80–100; 17/17). There was no significant difference in the PCR sensitivity according gender, age, location of skin lesion, and type of skin lesion.”

Discussion-

“Taken together, despite no significant difference, the detection rate of scabies by PCR tended to be higher than that of the microscopy examination, suggesting that the high sensitivity of scabies PCR.

In this study, the topographic distribution of skin lesions in patients with scabies presented similarly in a recent study [2]. The torso was the most common location involving scabies. The proportion of hand (43%) and genitalia (23%) was relatively higher than that in the recent study (36% and 7%) [2]. The possible explanation for this observation might be that this study mainly included symptomatic patients in the non-outbreak situation, unlike the recent study based on the nosocomial outbreaks [2]. It is worth to note that there are limited data on whether the sensitivity of the microscopic examination depends on the location of skin lesions. We found that there was no statistically significant difference in the scabies PCR sensitivity according to the locations of skin lesions. However, further studies are needed on this area.

There are some potential limitations to our study.”

**3. Results- Samples collected for microscopy and PCR were from the same place? Give details (Could be the reason for getting different results). Do you search only one sample or you repeated?? New statistical analyses could be required for lesion distribution and type.**

🡪 As the reviewer’s comments, we have further described the detail method of obtaining skin scrapings in the Method section of the revised manuscript as follows.

“Skin scrapings for patients with suspected scabies were obtained from clinically suspected lesions by an experienced nurse specialist for microscopic examination and scabies PCR simultaneously [13]. The specimens were collected at 10~15 sites for all delineated lesions. Skin scrapings for healthy volunteers were obtained from extensor sites of extremities, which were appropriate places in getting keratinous layer without skin lesions. Decision regarding the appropriate anti-scabies treatment was made based on the clinical features and microscopic results and the blinded results of the scabies PCR by the attending dermatologist. Only the results of scabies PCR and microscopic examination for the specimens obtained on the first day of diagnosis with scabies were included in the analysis. Written informed consent was obtained from all patients.”

As mentioned above, the torso (include groin) was the most common location in 27 patients (90%), followed by the extremities in 22 (73%) of the patients (S1 Fig). The scabies PCR sensitivities according skin lesions were 82% at extremities, 81% at torso, and 85% at extremities and torso, respectively (*p*=0.99). Of 30 patients with scabies, 23 had papule and 7 had other skin lesions such as nodule and excoriated patches. The PCR sensitivity was 83% vs. 71%, respectively (*p*=0.60). We have added this information in the Results section.

“The torso was the most common location in 27 patients (90%), followed by the extremities in 22 (73%), ~~the groin in 9 (30%),~~ and the genitalia in 7 (23%) of the patients. Approximately 43% of patients with scabies had the skin lesion in the hands (S1 Fig).”

“Thus, the specificity of the scabies PCR in no scabies controls was of 100% (95% CI=80–100; 17/17). There was no significant difference in the PCR sensitivity according gender, age, location of skin lesion, and type of skin lesion.”

**4. Results- For 222-225 "Of the 30 patients with scabies, 5 (17%) revealed negative results for microscopic examination, but positive results for scabies PCR". Which type of scabies? Confirmed, clinical, or suspected? "On the other contrary, 3 patients (10%) exhibited positive results for microscopic examination, but negative results for scabies PCR". Which type of scabies? Confirmed, clinical, or suspected?**

🡪 Five patients who had negative results for microscopic examination but positive results for scabies PCR included one patient with clinical scabies and four patients with suspected scabies. All three patients who had positive results for microscopic examination but negative results for scabies PCR were confirmed scabies according to IACS criteria. We have added this information the Results section as follows:

“Of the 30 patients with scabies, 5 (17%) revealed negative results for microscopic examination, but positive results for scabies PCR. They were composed of 1 patient with clinical scabies and 4 patients with suspected scabies. On the other hand, 3 patients (10%) exhibited positive results for microscopic examination, but negative results for scabies PCR.”

**5. Conclusions- Additional evaluation mentioned in the result section that should be discussed in discussion section.**

🡪 We have added additional information about the scabies PCR sensitivity according to the distribution of skin lesions in the Results section of the revised manuscript, and we have discussed that in the Discussion section of the revised manuscript.

Discussion-

“Taken together, despite no significant difference, the detection rate of scabies by PCR tended to be higher than that of the microscopy examination, suggesting that the high sensitivity of scabies PCR.

In this study, the topographic distribution of skin lesions in patients with scabies presented similarly in a recent study [2]. The torso was the most common location involving scabies. The proportion of hand (43%) and genitalia (23%) was relatively higher than that in the recent study (36% and 7%) [2]. The possible explanation for this observation might be that this study mainly included symptomatic patients in the non-outbreak situation, unlike the recent study based on the nosocomial outbreaks [2]. It is worth to note that there are limited data on whether the sensitivity of the microscopic examination depends on the location of skin lesions. We found that there was no statistically significant difference in the scabies PCR sensitivity according to the locations of skin lesions. However, further studies are needed on this area.

There are some potential limitations to our study.”

**6. Writing of the references should be checked**

🡪 As the reviewer’s comments, we have corrected the references according to the JOURNAL’s guidelines.

**Reviewer #2’s comment:**

**1. Methods- The study design was appropriate to address the stated objectives, with the novel use of IACS consensus criteria as ‘gold standard’, allowing sensitivity for PCR and microscopy to be calculated independently of each other. Additional information, and correction of some details is required, as follows:**

**1) Please double check the numbers in each of the categories throughout the study, as follows:**

**Line 62-65: The numbers of participants in each group is inconsistent with the total number of participants, as well as differing from the description given later in the paper. I.e. 43 suspected +14 other diseases+4 healthy = 61, not 57; 22 confirmed + 2 clinical + 6 suspected + 3 no scabies=33 suspected scabies cases, not 43.**

**Line 193-194: Here, the 14 participants without suspected scabies is broken down into 10 with onychomychosis and 4 healthy, inconsistent with the abstract in which the 4 healthy were stated as additional to the 14.**

**Line 195-196: As per the abstract, the numbers in each category of scabies do not add to the total stated with suspected scabies.**

**Line 198-200: With 17 controls (10 with onychomychosis, 3 with no scabies, 4 healthy volunteers) and 30 scabies diagnoses, it appears as though the total number of participants was 47 rather than 57.**

🡪 As the reviewer’s comments, “43” is typo-error. “33” is correct. A total of 47 participants, 33 with suspected scabies, 10 with onychomycosis, and 4 healthy volunteers were included. Of the 33 patients with suspected scabies, 22 were classified as confirmed scabies, 2 as clinical scabies, 6 as suspected scabies, and 3 as no scabies. A total of 17 participants including 3 with no scabies, 10 with onychomycosis, and 4 healthy volunteers were classified as no scabies control. We corrected each number and revised the manuscript as follows.

Abstract-

“A total of 47 participants, 33 with suspected scabies, 10 with unrelated diseases, and 4 healthy volunteers were enrolled. Of the 33 patients, 22 were classified as confirmed scabies, 2 as clinical scabies, 6 as suspected scabies, and 3 as no scabies.”

Results-

“A total of 47 participants including 33 patients with suspected scabies and 14 participants with an unrelated disease (10 patients with onychomycosis and 4 healthy volunteers) were enrolled in this study. Of the 33 patients with suspected scabies, 22 were classified as confirmed scabies, 2 as clinical scabies, 6 as suspected scabies, and 3 as no scabies (2 as drug eruption, 1 as another superficial fungal infection).”

“Skin scraping specimens from 10 patients with onychomycosis, 4 healthy donors without any other dermatologic condition, and 3 classified as no scabies by the IACS criteria were all negative for the PCR test.”

**2) Where were the skin scrapings taken from for the healthy volunteers with no scabies? To ensure comparability with the scrapings taken from suspected cases, the sampling sites should be similar. Please state the sampling strategy for the controls in the methods.**

🡪 We see the reviewer’s point. The skin scrapings for healthy volunteers were taken from the limbs (e.g. elbows and heels) because these areas were relatively appropriate place in getting scales in healthy volunteers without skin lesions. Of the 30 patients with scabies, 22 (73%) had skin lesions on the extremities and skin scraping was performed at these lesions. Therefore, the method to collect the samples from the location for skin scraping in patients and healthy volunteers was similar. We have added this information in the Method section of the revised manuscript as follows.

“As a first step, an experienced dermatologist delineated clinically suspicious lesions. Skin scrapings for patients with suspected scabies were obtained from clinically suspected lesions by an experienced nurse specialist for microscopic examination and scabies PCR simultaneously [13]. The specimens were collected at 10~15 sites for all delineated lesions. Skin scrapings for healthy volunteers were obtained from extensor sites of extremities, which were appropriate places in getting keratinous layer without skin lesions. Decision regarding the appropriate anti-scabies treatment was made based on the clinical features and microscopic results and the blinded results of the scabies PCR by the attending dermatologist.”

**3) Line 182-183: Could you please state that you have calculated ‘exact’ confidence intervals (your results in Table 1 appear to be consistent with this).**
🡪 Per the reviewer’s comment, we have added additional information about statistical analysis.

“The sensitivity and ~~specificity~~ of the scabies PCR was compared with those of microscopic examination in each clinical category group. Data are expressed as percent and Clopper-Pearson exact 95% CI. The linear-by-linear association test was used to examine the linear association between the level of diagnostic certainty and the sensitivity of the microscopic examination or the scabies PCR test.”

**2. Results- The results presented follow the outlined methods, with tables and figures clearly presented. There are minor corrections required to the numbers, with details provided under the methods section.**

🡪 As the reviewer’s comments, “43” is typo-error. “33” is correct. A total of 47 participants, 33 with suspected scabies, 10 with onychomycosis, and 4 healthy volunteers were included. Of the 33 patients with suspected scabies, 22 were classified as confirmed scabies, 2 as clinical scabies, 6 as suspected scabies, and 3 as no scabies. A total of 17 participants including 3 with no scabies, 10 with onychomycosis, and 4 healthy volunteers were classified as no scabies control. We corrected each number and revised the manuscript as follows.

Results-

“A total of 47 participants including 33 patients with suspected scabies and 14 participants with an unrelated disease (10 patients with onychomycosis and 4 healthy volunteers) were enrolled in this study. Of the 33 patients with suspected scabies, 22 were classified as confirmed scabies, 2 as clinical scabies, 6 as suspected scabies, and 3 as no scabies (2 as drug eruption, 1 as another superficial fungal infection).”

“Skin scraping specimens from 10 patients with onychomycosis, 4 healthy donors without any other dermatologic condition, and 3 classified as no scabies by the IACS criteria were all negative for the PCR test.”

**3. Conclusions- The conclusions are supported by the experimental results and the limitations of the study are clearly described. Further work required is described. A couple of sentences should be clarified, as follows:**

**1) Line 254-255: ‘clinical or suspected cases is more likely to be detected by the scabies PCR’ – this needs to be reworded. By design, clinical or suspected cases cannot be positive for microscopy – because if they were, then they would be confirmed cases. Therefore, it doesn’t make sense to compare the ability of the scabies PCR and microscopy to identify such cases. It would be better to discuss in terms of PCR having an ability to identify cases that could not be confirmed by microscopy.**

🡪 As the reviewer’s comments, we reworded these sentences in the revised manuscript as follows.

“However, given the high specificity of the scabies PCR in our study and previous consistent reports on the low sensitivity of the microscopic examination, the scabies PCR was considered to have an ability to identify cases that could not be confirmed by microscopic examination. Our findings support this hypothesis and showed that the scabies PCR test detected five additional patients (17%) that were not initially diagnosed by microscopic examination.”

**2) Line 274-275: Did you statistically test whether there was a difference in the sensitivity for PCR and for microscopy? From what I can see, you tested whether there was a difference for each of these methods across the different IACS categories, but didn’t test the methods against each other. Please change the wording to ensure it is clear whether or not a formal statistical test was undertaken.**

🡪 We see the reviewer’s point, we calculated the sensitivity of both scabies PCR test and microscopy assays for each clinical category according to the level of diagnostic certainty and investigated the linear association between the level of diagnostic certainty and the sensitivity of the microscopic assays or the scabies PCR, respectively. We also compared with the sensitivity of scabies PCR and microscopy assay in each clinical category group. We have revised this point in the Method and the Results section as follows.

Method-

“To investigate the diagnostic value of the PCR testing, we calculated the 95% confidence interval (CI) of the sensitivity and specificity of both PCR and microscopy assays for each clinical category according to the level of diagnostic certainty: confirmed; confirmed and clinical; and confirmed, clinical, and suspected scabies. The sensitivity ~~and specificity~~ of the scabies PCR was compared with those of microscopic examination in each clinical category group. Data are expressed as percent and Clopper-Pearson exact 95% CI. The linear-by-linear association test was used to examine the linear association between the level of diagnostic certainty and the sensitivity of the microscopic examination or the scabies PCR test.”

Results-

**Table 1. Comparison of diagnostic performance of the scabies PCR and microscopy in confirmed, clinical, and suspected scabies patients.**

|  |  | **Confirmed scabies**  **(n=22)** | **Confirmed + clinical**  **scabies (n=24)** | **Confirmed + clinical**  **+ suspected scabies**  **(n=30)** | **No scabies**  **(n=17)^a^** |
| --- | --- | --- | --- | --- | --- |
| **PCR** | Positive (n) | 19 | 20 | 24 | 0 |
|  | Negative (n) | 3 | 4 | 6 | 17 |
|  | Sensitivity (%, 95% CI)^b^ | 86 (65–97)^d^ | 83 (63–95)^e^ | 80 (61–92)^f^ |  |
|  | Specificity (%, 95% CI) |  |  |  | 100 (80–100) |
| **Microscopy** | Positive (n) | 22 | 22 | 22 | 0 |
|  | Negative (n) | 0 | 2 | 8 | 17 |
|  | Sensitivity (%, 95% CI)^c^ | 100 (85–100)^d^ | 92 (73–99)^e^ | 73 (54–88)^f^ |  |
|  | Specificity (%, 95% CI) |  |  |  | 100 (80–100) |

^a^“No scabies” included 13 skin scrap specimens from patients with alternative diagnosis and four skin scrap specimens from healthy volunteers.

^b^Difference in the sensitivity of the PCR test between the three groups was not statistically significant (*p*=0.59).

^c^Difference in the sensitivity of the microscopic examination between the three groups was statistically significant (*p*=0.006).

^d^Difference in the sensitivity between PCR and the microscopic examination in the patients with confirmed scabies was not statistically significant (*p*=0.23).

^e^Difference in the sensitivity between PCR and the microscopic examination in the patients with confirmed and clinical scabies was not statistically significant (*p*=0.67).

^f^Difference in the sensitivity between PCR and the microscopic examination in the patients with confirmed, clinical, and suspected scabies was not statistically significant (*p*=0.54).

“The sensitivity of the scabies PCR test was 86% (95% CI=65–97; 19/22), 83% (95% CI=63–95; 20/24), and 80% (95% CI=61–92; 24/30) in confirmed scabies; confirmed and clinical scabies; and confirmed, clinical, and suspected scabies, respectively (*p=*0.59). As the level of diagnostic certainty decreased, the sensitivity of the scabies PCR also decreased, but there was no statistically significant difference between the groups. The sensitivity of scabies PCR test was slightly higher than that of the microscopic examination but there was no statistically significant differences between these tests in patients with confirmed, clinical, and suspected scabies (80% vs 73%, *p*=0.54). Of the 30 patients with scabies, 5 (17%) revealed negative results for microscopic examination, but positive results for scabies PCR.”

**4. Minor corrections and suggested re-phrasing:**

**1) Line 28: ‘Sarcoptes’, not ‘Scarcoptes’; 2) Line 30: ‘proposed by the International…’; 3) Line 31: ‘tests’ not ‘test’; 4) Line 32: as no specific PCR test has yet been mentioned, ‘this in-house…’ does not make sense; 5) Line 62: ‘scabiei’, not ‘scabies’; 6) Line 74: delete ‘is’; 7) Line 76: ‘result in various skin eruptions…’; 8) Line 87: ‘tests’, not ‘test’**

**9) Line 145: ‘were performed’; 10) Line 158: ‘scraping’, not ‘scrapping’; 11) Line 197-198 seems to simply repeat the preceding sentence.; 12) Line 203: 10/30 = 33%, not 30%; 13) Line 214: for confirmed, clinical and suspected, it should be 22/30 (73% is correct), not 24/30; 14) Line 224: ‘on the other contrary’ sounds strange – how about ‘on the other hand’?; 15) Line 261: ‘The authors’, not ‘they’**

🡪 As the reviewer’s comments, we have corrected the typo-error in the revised manuscript.

Author summary-

“We hypothesized that polymerase chain reaction (PCR) detection of *Sarcoptes scabiei* DNA directly has higher sensitivity than microscopic examination.”

“Recently, clinical consensus criteria have been proposed by the International Alliance for the Control of Scabies (IACS) to overcome … unrelated disease according to the criteria of the IACS. …between 86% and 80% as the level of diagnostic certainty by the IACS criteria decreased, … 100% to 73% as the level of diagnostic certainty by the IACS criteria decreased.”

“.. to overcome the low sensitivities of conventional diagnostic tests for scabies.”

“We thus evaluated the diagnostic capability of ~~this~~ in-house real-time PCR for the diagnosis of scabies from skin scraping”

Abstract-

“PCR was performed on the skin scrapings to target the cytochrome c oxidase subunit 1 (cox1) gene of *Sarcoptes scabiei*.”

Introduction-

“Scabies ~~is~~ caused by *Sarcoptes scabiei* mites, is a highly contagious parasitic disease …”

“Infections by scabies mite results in various skin eruptions consisting of …”

“Recently, clinical consensus criteria have been proposed by the International Alliance for the Control of Scabies (IACS) to overcome the low sensitivities of conventional diagnostic tests for scabies.”

“… the previously used criteria of the IACS.”

Method-

“Multiplex real-time PCR assays to detect *S. scabiei* were performed …”

“Fig 1. Detection of *Sarcoptes scabiei* DNA by polymerase chain reaction (PCR). DNA was purified from skin scrap~~p~~ing samples …”

Results-

“Of the 33 patients with suspected scabies, 22 were classified as confirmed scabies, 2 as clinical scabies, 6 as suspected scabies, and 3 as no scabies (2 as drug eruption, 1 as another superficial fungal infection). ~~Diagnosis of scabies (n=30) was made at one of the three levels as follows: confirmed (n=22), clinical (n=2), suspected (n=6). In addition,~~ A total of 17 subjects including 3 with no scabies, 10 with onychomycosis, and 4 healthy volunteers were classified as no scabies control.”

“Thirteen of these patients (43%) had a history of hospitalizations and 10 (33%) had a history of hospital visits …”

“The sensitivity of the microscopic examination was 100% (95% CI=85–100; 22/22), 92% (95% CI=73–99; 22/24), and 73% (95% CI=54–88; 22/30) in confirmed scabies; confirmed and clinical scabies; and confirmed, clinical, and suspected scabies, respectively (*p=*0.006).”

“On the other hand, 3 patients (10%) exhibited positive results for microscopic examination, but negative results for scabies PCR.”

Discussion-

“Possible explanations for this observation are that patients with clinical or suspected scabies by the IACS criteria …”

“The authors performed sequencing of *cox1* gene obtained in mites from 60 humans and showed that mites could belong to different clades genetically even if they were collected in the same area.”
